# Is Hepatectomy Necessary in Dealing with Left Hepatolithiasis with Intrahepatic Duct Stricture?

**DOI:** 10.1155/1997/87823

**Published:** 1997

**Authors:** Dominique Franco

**Affiliations:** Groupe de Recherche sur la Chirurgie du Foie et de l'Hypertension Portale Hôpital Antoine Béclére Clamart 92141 France

## Abstract

*Background*: Hepatolithiasis with intrahepatic biliary strictures, more common in Southeast Asia than elsewhere, remains a difficult problem to manage. Hepatic resection has recently been advocated as one of the treatment modalities for hepatolithiasis; however, this procedure is not without risk. This study was designed to achieve complete clearance of the stones, eliminate bile stasis, and avoid the potential risks of hepatic resection in the patient with hepatolithiasis and intrahepatic biliary stricture.

*Methods*: In this prospective clinical trial 13 patients with retained left hepatolithiasis and intrahepatic biliary strictures were included. All the patients met the following criteria: (1) initial surgical procedure for hepatolithiasis, (2) normal gross findings of the left liver, and (3) no obvious clinical evidence of an associated intrahepatic cholangiocarcinoma. After the operation they underwent matured T-tube tract ductal dilatation with percutaneous transhepatic cholangioscopy tube stenting. Choledoschoscopic electrohydraulic lithotripsy was used in five patients after dilatation when impacted or large stones were encountered.

*Results*: Complete clearance of the stones was achieved in these 13 patients. One patient had fevers develop after ductal dilatation, and another patient had mild hemobilia after electrohydraulic lithotripsy. Both recovered uneventfully with conservative treatment. These successfully treated patients remain well, with a mean follow-up period of 20 months.

*Conclusions*: Postoperative matured T-tube tract ductal dilatation and stenting, combined with endoscopic electrohydraulic lithotripsy when indicated, is an effective and safe alternative to hepatic resection for selected left hepatolithiasis with intrahepatic biliary stricture.


trol variceal bleeding before liver transplantation. *Annals of Internal Medicine*, **116**, 304–306.
[20] Rossle, M., Haag, K., Ochs, A., Sellinger, M., Noldge, G., Perarneu, J., Berger, E., Blum, V., Gabelmann, A. and Hauenstein, K. (1994). The trasjugular intrahepatic portosystemic stent shunt procedure for variceal bleeding. *New England Journal of Medicine*, **330**, 165–171.
[21] LaBerge, J.M., Ring, E., Gordon, R., Lake, J., Doherty, M., Somberg, K., Roberts, J. and Ascher, N. (1993). Creation of transjugular intrahepatic portosystemic shunts with the wallstent endoprosthesis: Results in 100 patients. *Radiology*, **187**, 413–420.
[22] Shiman, M., Jeers, L., Hoofnagle, J. and Tralka, T. (1995). The role of transjugular intrahepatic shunt (TIPS) for treatment of portal hypertension and its complications. National Digestive Diseases Advisory Board. *Hepatology*, **22**, 1591–1597.
[23] Millis, J.M., Martin, P., Gomes, A., Shaked, A., Coliquhoun, S.D., Jurim, O., Goldstein, L. and Busuttil, R.W. (1995). Transjugular intrahepatic portosystemic shunts: Impact on liver transplantation. Liver Transplantation and Surgery, **1**, 229–233.
[24] Gores, G.J., Wiesner, R.H., Dickson, E.R., Zinmeister, A.R., Jorgensen, R.A. and Langworthy, A. (1989). Prospective Evaluation of esophageal varices in primary biliary cirrhosis. *Gastroenterology*, **96**, 1552–1559.
[25] Graham, D.Y. and Smith, J.L. (1981). The course of patients after variceal hemorrhage. *Gastroenterology*, **80**, 800–809.
[27] Wood, P., Shaw, B. and Rikkers, L. (1990). Liver transplantation for variceal hemorrhage. Surgical Clinics of North America, **70**, 449–461.
[28] Bismuth, H., Adam, R., Mathur, S. and Sherlock, D. (1990). Options for elective treatment of portal hypertension in cirrhotic patients in the transplantation era. *American Journal of Surgery*, **160**, 105–110.
[29] Coldwell, D.M., Ring, E.J., Rees, C.R., Zemel, G., Darcy, M.D., Haskal, Z.J., McKusick, M.A. and Greenfield, A.J. (1995). Multi-center investigation of the role of transjugular intrahepatic portosystemic shunt in the management of portal hypertension. *Radiology*, **196**, 335–340.
[30] Rubin, R.A., Haskal, Z.J., O'Brien, C.B., Cope, C. and Brass, C.A. (1995). Transjugular intrahepatic portosystemic shunting: Decreased survival for patients with high APACHE II scores. *American Journal of Gastroenterology*, **90**, 556–563.

Thomas W. Faust M. D.
Michael F. Sorrell M. D.
University of Nebraska Medical Center
Liver Transplant Program

Michael F. Sorrell, M. D.
University of Nebraska Medical Center
Liver Transplant Program
600 S. 42nd Street
Omaha, Nebraska 68198–3280

### Is Hepatectomy Necessary in Dealing with Left Hepatolithiasis with Intrahepatic Duct Stricture?

### ABSTRACT

*Sheen-Chen, S-M., Cheng, Y-F., Chou, F-F.* and *Lee, T-Y. (1995). Ductal dilatation and stenting make routine hepatectomy unnecessary for left hepatolithiasis with intrahepatic biliary stricture. Surgery; 117: 32–36.*

*Background:* **Hepatolithiasis with intrahepatic biliary strictures, more common in Southeast Asia than elsewhere, remains a difficult problem to manage. Hepatic resection has recently been advocated as one of the treatment modalities for hepatolithiasis; however, this procedure is not without risk. This study was designed to achieve complete clearance of the stones, eliminate bile stasis, and avoid the potential risks of hepatic resection in the patient with hepatolithiasis and intrahepatic biliary stricture.**
*Methods:* **In this prospective clinical trial 13 patients with retained left hepatolithiasis and intrahepatic biliary strictures were included. All the patients met the following criteria: (1) initial surgical procedure for hepatolithiasis, (2) normal gross findings of the left liver, and**

**(3) no obvious clinical evidence of an associated intrahepatic cholangiocarcinoma. After the operation they underwent matured T-tube tract ductal dilatation with percutaneous transhepatic cholangioscopy tube stenting. Choledoschoscopic electrohydraulic lithotripsy was used in five patients after dilatation when impacted or large stones were encountered.**
***Results:*** **Complete clearance of the stones was achieved in these 13 patients. One patient had fevers develop after ductal dilatation, and another patient had mild hemobilia after electrohydraulic lithotripsy. Both recovered uneventfully with conservative treatment. These successfully treated patients remain well, with a mean follow-up period of 20 months.**
***Conclusions:*** **Postoperative matured T-tube tract ductal dilatation and stenting, combined with endoscopic electrohydraulic lithotripsy when indicated, is an effective and safe alternative to hepatic resection for selected left hepatolithiasis with intrahepatic biliary stricture. (SURGERY 1995; 117:32–36.)**

*Keywords*: Intrahepatic gallstones, hepatolethiasis, hepatectomy, bile duct dilatation

### PAPER DISCUSSION

The treatment of intrahepatic gallstones remains controversial. In South East Asia where this disease is frequent a clear trend has emerged in favor of endoscopic destruction and/or removal of intrahepatic stones associated with dilatation and/or stenting of ductal strictures [1–5]. These procedures have been greatly facilitated by the development of biliary imaging and of cholangioscopy and cholangioscopic electrohydraulic or laser lithotripsy. Intrahepatic bile ducts may be approached during operation or postoperatively through several routes such as dilated T-tube tract [5] or dilated percutaneous transhepatic drainage tract [3], or the jejunal limb of a hepaticocutaneous jejunostomy [1,2]. Complete stone clearance is achieved in 75% to 90% of patients and the rate of stone recurrence is about 15% to 20%. It is more frequent when it has not been possible to dilate all bile duct strictures (3). In the work published by Sheen-Chen *et al.* the authors show that cholangioscopic stone retrieval with stricture stenting is an excellent treatment in patients with hepatolithiasis localized in the left lateral lobe. They therefore suggest that liver resection has no more rationale in hepatolithiasis, even in this group of patients in whom it was the best indication.

Such an assertion might be slightly provocative and represent an oversimplification of the therapeutic approach of intrahepatic stones. Firstly the cholangioscopic protocol is not as simple as argued by the authors. It may require a number of therapeutic sessions in a single patient in order to dilate and/or stent all bile duct strictures and to remove all stones. Despite use of semi-rigid dilators endoscopic maneuvers in patients with dilated bile ducts and infection are not without risks. Two patients of their series experienced complications. Figures were even higher in other series [2], and the mortality rate was 3.8% in the series of Chijiiwa *et al.* [4]. While the T-tube route exposes to less risks than the transhepatic one it can be applied only in patients who underwent surgery as a first procedure and presented with postoperative retained stones.

Secondly the late follow-up may not be as good as claimed here. In another series in Taiwan only 46% of the group of patients treated by percutaneous transhepatic cholangioscopy were free of symptoms with a follow-up of 4 to 10 years [3]. There were early failures in 17% of patients and recurrent stones and/or cholangitis after primary clearance in 38%. Rates of recurrence between 11% and 15% despite complete stone clearance have been reported by others [1,4]. Recurrence is mainly determined by the success or failure to dilate biliary strictures and to remove distal stones. However late complications also occur when the liver is chronically damaged by long-standing cholestasis and cholangitis [4].

Thirdly cholangiocarcinoma occurs in about 10% of patients with hepatolithiasis [6]. Although there is no direct evidence that stones themselves are responsible for the development of carcinoma, mechanical irritation from the stones, bile stasis and infection may be important etiologic factors leading to adenomatous epithelial hyperplasia and bile duct carcinoma. Cholangiocarcinoma may occur a long time after therapeutic clearance of stones as shown by Chijiiwa *et al.* in a cohort of 109 treated patients [7]. In 7 patients with onset of cholangiocarcinoma during follow-up, 3 had no recurrent stones. This means that a potential risk of cholangiocarcinoma remains in these patients whatever the success of the initial procedure. Also when analyzing factors predisposing to cholangiocarcinoma, Kubo *et al.* [6] found that the localisation of stones predominantly in the left lateral lobe is associated with a greater risk of cancer than other locations. Therefore these patients in whom Sheen-Chen and al recommend conservative treatment might be those who need more. A certain number of investigations might help in the early diagnosis of hyperplasia and cholangiocarcinoma such as histochemical analysis of the mucosa or dosage of tumor markers in the bile. This may further pinpoint patients is whom liver resection is mandatory.

The risk of liver resection in patient with hepatolithiasis is not high. The hospital mortality averages 2% [8,9]. Left lateral lobectomy especially are low risk surgical procedures. Postoperative sepsis may be more frequent than following resection for a liver tumor because of leakage of infected bile in the operative field [8] but its rate is decreased by intraoperative antibiotics [10]. As observed with other treatments late results after hepatectomy for hepatolithiasis largely depend upon the complete relief of stones and of ductal anomalies. The best results are obtained in patients with localized lesions which can be totally resected, leaving a normal remnant liver. Such patients with localized stones and ductal anomalies are not as uncommon as usually admitted. They did represent about two thirds of 137 patients with hepatolithiasis in the series of Queen Mary Hospital, Hong-Kong [1], and of 85 patients in the series of Fukuoka, Japan [4]. In the West the localized cases may even be more common [10]. Chijiiwa *et al.* have shown that results after resection are better in patients without than in those with previous biliary drainage [9]. This suggests that, whenever indicated, liver resection should be performed as the first therapeutic procedure.

Pathogenesis of hepatolithiasis is not yet completely understood. The formation of stones takes place because of bile stasis in dilated bile ducts and is enhanced by infection, changes in bile composition and/or metabolic derangement of the liver [11]. The role of bile duct strictures is not clear. They do not seem to be responsible for bile duct dilatation but might rather result secondarily from irritation by stones and cholangitis [12] and perpetuate bile stasis. Intrahepatic bile ducts dilatation might be congenital and their distribution be segmental. With time stones formed in the dilated ducts may migrate into the common bile duct and/or spread through the whole liver. Modern procedures of liver and biliary investigations such as ultrasonograhy, enhanced CT scan and cholangio-MRI, allow early recognition of hepatolithiasis in patients with cholangitis and precise localization of the disease. This results in avoidance of unsuited therapeutic procedures and in the choice of the most adapted treatment at first. We believe that when biliary anomalies (dilatation and stones) are restricted to one liver area the best treatment offered to patients and together the safest and least expansive is liver resection which results in complete and definitive cure [10]. When biliary anomalies are disseminated throughout the liver relief of bile stasis by removal of stones and dilatation of strictures remains the most appropriate treatment. In these cases when liver damage is severe, liver transplantation should not be excluded. Readers in South East Asia might not agree which such conclusions. While hepatolithiasis is much more frequent in that part of the world than in the West it might not be so different.

### References

[1] Fan, S.T., Choi, T.K., Lo, C.M., Mok, F.P.T., Lai, E.C.S. and Wong, J. (1991).. Treatment of hepatolithiasis: Improvement of result by a systematic approach. *Surgery*, **109**, 474–80.

[2] Jeng, K.S., Yang, F.S., Chiang, H.J. and Ohita, I. (1992).. Bile duct stents in the management of hepatolithiasis with large-segment intrahepatic biliary strictures. *Br J Surg*, **79**, 663–6.

[3] Jan, Y.Y. and Chen, M.F. (1995). Percutaneous transhepatic cholangioscopic lithotomy for hepatolithiasis: long-term results. *Gastrointes Endosc*, **42**, 1–5.

[4] Chijiiwa, K., Yamashita, H., Yoshida, J., Kuroki, S. and Tanaka, M. (1995). Current management and long-term prognosis of hepatolithiasis. *Arch Surg*, **130**, 194–7.

[5] Cheng, Y.F., Chen, T.Y., Ko, S.F., Huang, C.C., Huang, T.L., Weng, H.H., Lee, T.Y. and Sheen-Chen, S.M. (1995). Treatment of postoperative residual hepatolithiasis after progressive stenting of associated bile duct strictures through the T-tube tract. *Cardiovasc Intervent Radiol*, **18**, 77–81.

[6] Kubo, S., Kinoshita, H., Hirohashi, K. and Hamba, H. (1995). Hepatolithiasis associated with cholangiocarcinoma. *World J Surg*, **19**, 637–41.

[7] Chijiiwa, K., Ichimiya, H., Kuroki, S., Koga, A. and Nakayama, F. (1993). Late development of cholangiocarcinoma after the treatment of hepatolithiasis. *Surg Gynecol Obstet*, **177**, 279–82.

[8] Fan, S.T., Lai, E.C.S. and Wong, J. (1993). Hepatic resection for hepatolithiasis. *Arch Surg*, **128**, 1070–4.

[9] Chijiiwa, K., Kameoka, N., Komura, M., Yamasaki, T., Noshiro, H. and Nakano, K. (1995). Hepatic resection for hepatolithiasis and long-term results. *J Am Coll Surg*, **180**, 43–8.

[10] Borgonovo, G., Vons, C., Smadja, C., Grange, D. and Franco, D. (1996). Liver resection: a treatment of choice in patients with primary localized intrahepatic gallstones. *HPB Surgery*, **9**(S), 154.

[11] Kim, M.H., Sekijima, J. and Lee, S.P. (1995). Primary intrahepatic stones. *Am J gastroenterol*, **90**, 540–8.

[12] Matsumoto, Y., Fujii, H., Yoshioka, M., Sekikawa, T., Wada, T., Yamamoto, M., Eguchi, H. and Sugahara, K. (1986). Biliary strictures as a cause of primary intrahepatic bile duct stones. *World J Surg*, **10**, 867–75.

Dominique Franco, M.D.
Groupe de Recherche sur la Chirurgie du Foie
et de l'Hypertension Portale
Hôpital Antoine Béclére
92141 Clamart
France

